# The eNOS isoform exhibits increased expression and activation in the main olfactory bulb of nNOS knock-out mice

**DOI:** 10.3389/fncel.2023.1120836

**Published:** 2023-03-16

**Authors:** David Pérez-Boyero, Carlos Hernández-Pérez, Jorge Valero, Valeria Lorena Cabedo, José Ramón Alonso, David Díaz, Eduardo Weruaga

**Affiliations:** ^1^Institute for Neuroscience of Castilla and León (INCYL), Universidad de Salamanca, Salamanca, Spain; ^2^Institute of Biomedical Research of Salamanca (IBSAL), Salamanca, Spain

**Keywords:** nitric oxide, nitric oxide synthase, olfaction, olfactory bulb, main olfactory bulb, plasticity

## Abstract

The main olfactory bulb (MOB) is a neural structure that processes olfactory information. Among the neurotransmitters present in the MOB, nitric oxide (NO) is particularly relevant as it performs a wide variety of functions. In this structure, NO is produced mainly by neuronal nitric oxide synthase (nNOS) but also by inducible nitric oxide synthase (iNOS) and endothelial nitric oxide synthase (eNOS). The MOB is considered a region with great plasticity and the different NOS also show great plasticity. Therefore, it could be considered that this plasticity could compensate for various dysfunctional and pathological alterations. We examined the possible plasticity of iNOS and eNOS in the MOB in the absence of nNOS. For this, wild-type and nNOS knock-out (nNOS-KO) mice were used. We assessed whether the absence of nNOS expression could affect the olfactory capacity of mice, followed by the analysis of the expression and distribution of the NOS isoforms using qPCR and immunofluorescence. NO production in MOB was examined using both the Griess and histochemical NADPH-diaphorase reactions. The results indicate nNOS-KO mice have reduced olfactory capacity. We observed that in the nNOS-KO animal, there is an increase both in the expression of eNOS and NADPH-diaphorase, but no apparent change in the level of NO generated in the MOB. It can be concluded that the level of eNOS in the MOB of nNOS-KO is related to the maintenance of normal levels of NO. Therefore, our findings suggest that nNOS could be essential for the proper functioning of the olfactory system.

## Introduction

The sense of olfaction is managed by a chemosensory system that allows animals to identify several odorant molecules of different chemical nature. Thanks to this sense of smell, animals can detect essential signals necessary for their survival such as interspecific and intraspecific indicators, food, toxins, and predators, among others (Agustín-Pavón et al., 2009; Sanchez-Andrade and Kendrick, 2009). The olfactory system is divided into two components (Shipley and Ennis, 1996), separated both functionally and anatomically. On the one hand, there is the main olfactory system that is responsible for capturing and transmitting signals produced by volatile odorants. It contains the main olfactory bulb (MOB), the nerve structure on which this work will focus, and which deserves a more detailed description later on. On the other hand, there is the accessory or vomeronasal olfactory system which is involved in processing pheromonal information. In this second system, the accessory olfactory bulb (AOB) -adjacent to the MOB- is the first processing station for pheromonal signals.

This study focuses on the MOB, which is the first signal-processing station of the olfactory system. In rodents, the MOB is a neural structure that comprises the most rostral region of the brain (Díaz et al., 2013). Succinctly, the MOB is comprised mainly of input and output fibers, projecting neurons, and local interneurons distributed in seven concentric layers. In addition, the MOB presents highly complex neural circuits as well as a wide variety of neurotransmitters that allow the precise modulation of sensory information (McClintock et al., 2009; Yand et al., 2013). In this work, we have focused our attention on one specific neurotransmitter, nitric oxide (NO), with unusual physicochemical properties.

Nitric oxide is a gaseous, highly hydrophobic, and diffusing molecule present in small amounts in most organisms (Lourenço et al., 2017). In biological fluids, it reacts rapidly with H_2_O and O_2_, forming its main active metabolites: nitrates (NO_3_^–^) and nitrites (NO_2_^–^) (Hunt and Navalta, 2012; Asiimwe et al., 2016). NO is synthesized by the action of nitric oxide synthase (NOS) by converting L-arginine to L-citrulline with the help of NADPH acting as a cofactor (Chen et al., 2004; Sabat et al., 2013). Currently, three types of synthases are known: neuronal NOS (nNOS), inducible or immune NOS (iNOS), and endothelial NOS (eNOS) (Hardingham et al., 2013); the genes of all three are located on different chromosomes. Although the isoforms have the same catalytic properties, they do have some distinct characteristics. The first difference is in the cells in which they are expressed. While nNOS is mainly expressed in neurons, iNOS appears mainly in macrophages and microglial cells, and eNOS is generally expressed in endothelial cells. Likewise, their expression patterns are also dissimilar; nNOS and eNOS are expressed constitutively whereas iNOS is expressed only through induction, generally after damage or pathological processes (Zhou and Zhu, 2009; Iwakiri, 2015; Oliveira-Paula et al., 2016; Zhu et al., 2016). Lastly, NO itself also has distinct functions, depending on the NOS species through which it is synthesized. The nitric oxide produced by nNOS acts as a neurotransmitter or neuromodulator, which is involved in important processes such as long-term potentiation and long-term depression (Asiimwe et al., 2016). However, the NO synthesized by iNOS plays a role in defense, functioning as an immunomodulatory substance, while the NO produced by eNOS acts as an efficient vasodilator that enables adequate blood flow to different tissues (Zhou and Zhu, 2009; Steinert et al., 2010; Iwakiri, 2015; Oliveira-Paula et al., 2016; Zhu et al., 2016). Although the site of expression and the main function of the NOS isoforms are those already mentioned, there are situations or environments in which these functions change (Zhu et al., 2003; Khan et al., 2012; Muñoz et al., 2015; Zhang et al., 2018). For this reason, we consider the plasticity of these enzymes in both physiological and pathological situations to be worthy of further research.

Remarkably, the three NOSs coexist in the central nervous system (Roskams et al., 1994; Zhou and Zhu, 2009; Steinert et al., 2010; Oliveira-Paula et al., 2016). In particular, the MOB presents an extremely high level of NO production, mainly due to the activity of nNOS, but also to the activity of iNOS and eNOS, depending on the physiological situation or the microenvironment (Bolaños and Almeida, 1999; Sung et al., 2014; Kozlov et al., 2017). Also, NO synthesis by different cell types has been observed in all seven layers of the MOB (McClintock et al., 2009; Yand et al., 2013). In the case of iNOS, labeling experiments have localized its expression mainly in cells associated with the immune system residing in the MOB, especially in microglia (Herbert et al., 2012; Zou et al., 2014), while the expression of eNOS has been found in blood vessels irrigating this structure (Kishimoto et al., 1993; Yand et al., 2013).

Several studies have provided evidence regarding the level of plasticity of the MOB (Díaz et al., 2009, 2017; Eckmeier and Shea, 2014), as well as the associated role of NOS in this process (Zhu et al., 2003; Khan et al., 2012; Muñoz et al., 2015; Zhang et al., 2018), especially under pathophysiological or abnormal conditions. Curiously, and as far as we know, there are no studies that directly relate the plasticity of the MOB and NO functions to the plasticity of NOS. Therefore, in the present study, the functions of NO and plasticity in the MOB have been evaluated in the absence of the main bulbar synthase, nNOS. For this purpose, olfactory behavior tests were conducted to check whether the loss of nNOS compromised the olfactory capacity of mice. In addition, any possible alteration in the expression of the other two NOS isoforms (iNOS and eNOS) was assessed in the MOB. And finally, the activity level of the different NOS isoforms and the total amount of NO produced were analyzed to determine whether the lack of nNOS could cause a possible compensatory modification in the production of NO by the two remaining isoforms.

## Materials and methods

### Animals

Knock-out mice for the NOS1 of the B6: 129S4-Nos1tm1Plh strain (The Jackson Laboratory, Bar Harbor, ME, USA) were used. To obtain these KO mice, the first exon of the *Nos1* gene was replaced by the neomycin-resistance gene, thus removing the first 159 amino acids of the functional protein NOS1 and preventing its production (Huang et al., 1993; Muñoz-Castañeda et al., 2014; Díaz et al., 2015). These mice were also crossed with wild-type animals of the 129 strain (The Jackson Laboratory) to obtain heterozygous mice, which were also crossed to finally obtain both wild-type (+/+) and KO (*Nos1*^–^/*Nos1*^–^) mice. These animals were used as breeders (wild-type or KO couples) of the animals used in this work. Following the original institutional guidelines,^1^ only these breeders were genotyped, and the genetic analysis of the offspring was not necessary. Subsequently, the male mice between the ages of 60 and 80 postnatal days (P60-P80) were used to avoid the effect of female hormones in the physiology of the MOB (Díaz et al., 2017). Animals were housed at the animal facility of the University of Salamanca at constant temperature and relative humidity, with a 12/12 h photoperiod, and were fed *ad libitum* with water and special rodent chow (Rodent toxicology diet, B&K Universal G.J., S.L., Barcelona, Spain). All animals were maintained, manipulated, and sacrificed in accordance with current European (2010/63/UE and Recommendation 2007/526/CE) and Spanish legislation (Law 32/2007 and RD 53/2013). The Bioethics Committee of the University of Salamanca approved the experiments carried out in this work (Reference number: #00613).

nNOS-KO mice were genotyped as they do not show a phenotype that allows them to be differentiated from their wild-type counterparts. For this purpose, a small tail sample was taken from each mouse before weaning and the cells were lysed for DNA extraction. PCR was then performed using primers specific to an exon of nNOS [IMR 0406 (sense) TCAGATCTGATCCGAGGAGG and IMR 0407 (antisense) TTCCAGAGCGCTGTCATAGC] and primers specific to the neomycin gene [IMR 0013 (sense) CTTGGG TGGAGAGGCTATTC and IMR 0014 (antisense) AGGTGAGATGACAGGAGATC] (De Brito Robalo et al., 2020). The products obtained were visualized on a 3% (w/v) agarose gel after electrophoresis.

### Behavior analyses

The buried food test, a simple method described and validated in previous works (Yang and Crawley, 2009), was used to check the ability of nNOS-KO mice to smell volatile odors and to assess whether the absence of nNOS compromised their olfactory capacity. To do this, 24 h before the test, both wild-type and nNOS-KO mice (*n* = 8 for each group) were weighed and deprived of food. One hour before the test was carried out, the mice were taken to the behavioral test room to acclimatize them to the environmental conditions. Again, the mice were weighed to ensure they had not lost more than 10% of their body weight. If this would have been the case, they would not have been used in the test as this measure is one of the guidelines to be followed when conducting the olfactory test for buried food (Yang and Crawley, 2009). Later, a piece of food (Rodent toxicology diet, B&K Universal G.J., S.L., Barcelona, Spain) was randomly placed in one of the corners of the cage where the experiment was carried out. Finally, each mouse was introduced into the center of the cage, and its behavior and the time it took for the mouse to find the food was recorded using a camera (Leica; Wetzlar, Germany). The food was considered to be discovered once the mouse had dug the pellet up and held it with its two front legs. Each mouse performed the test only once to avoid the interference of possible learning or habituation to the test (Yang and Crawley, 2009).

Additionally, we used the recordings of the mice to assess other behavioral parameters related to movement such as (1) the number of times the mouse approached the location in which the pellet was placed, (2) the number of uprisings, and (3) the number of grooming events.

### Gene analysis

Due to the absence of the nNOS in the mutant background, and to check for any possible compensatory expression of the other synthase genes, iNOS and eNOS gene expression was measured by quantitative polymerase chain reaction (qPCR) in MOB tissue. For this purpose, another set of mice (*n* = 4 for each group) was sacrificed by cervical dislocation and decapitation. Then, the MOB of each mouse was extracted and homogenized in a lysis buffer for extracting RNA using a kit (Thermo Fisher Scientific; Waltham, MA, USA). The RNA obtained was subjected to reverse transcription using a master mix comprising 10X buffer, 25 dNTP Mix (100 mM), 10X RT primers, MultiScribe reverse transcriptase, and nuclease-free H_2_O (Thermo Fisher Scientific).

Finally, the samples were subjected to qPCR in a QuantStudio 7 Flex Real-Time PCR System (Thermo Fisher Scientific), and gene expression was analyzed using QuantStudio™ Real-Time PCR software (v1.7.1, Thermo Fisher Scientific).

The oligos used for detecting iNOS (Thermo Fisher Scientific) were forward primer 5′-CTTTGCCACGGACGAGAC-3′ and reverse primer 5′-AACTTCCAGTCATTGTACTCTGAGG-3′, and for eNOS (Thermo Fisher Scientific) forward primer 5′-ATCCAGTGCCCTGCTTCA-3′ and reverse primer 5′-GCAGGGCAAGTTAGGATCAG-3′. GADPH (Thermo Fisher Scientific) was used as the housekeeping gene. The oligos used for this gene were forward primer 5′-GCCTATGTGGCCTCCAA-3′ and reverse primer 5′-GTGTTGGGTGCCCCTAGTTG-3′.

### Histological analyses

The mice destined for histological analyses (*n* = 4 or *n* = 5 for each group) were anesthetized and sacrificed by intracardiac perfusion using a peristaltic pump. The blood was initially washed with an isotonic saline solution (0.9% w/v), and then the Somogyi’s fixative without glutaraldehyde (4% paraformaldehyde and 0.2% picric acid in phosphate buffer, 0.1 M, pH 7.4, PB) was infused for 15 min.

The brains were dissected out and post-fixed in the same fixative solution for 2 h at continuous rotatory shaking. Then, the tissue blocks were cryo-protected with a solution of 30% (w/v) sucrose in 0.1 M PB and the MOBs were cut at 40-μm-thick coronal sections in a sliding microtome (Jung SM 2000, Type C blade, Leica Instruments, Nussloch, Germany) attached to a “Frigomobil” freezing unit.

The immunohistochemistry technique was applied to analyze the expression of the three NOS isoforms. For this purpose, the tissue sections were incubated in a mixture containing 5% donkey serum (v/v, Vector Laboratories, Burlingame, CA, USA), 0.2% triton X-100 (v/v, Probus S.A., Barcelona, Spain), and either a sheep anti-nNOS (1:10000 v/v, kindly provided by Dr. Emson and Dr. Charles, Cambridge, UK), mouse anti-iNOS (1:150 v/v, Abcam, Cambridge, UK) or mouse anti-eNOS (1:150 v/v; BD Transduction Laboratories, San Jose, California, USA) antibodies, in phosphate-buffered saline (pH 7.4; PBS), at 4°C for 24 h (nNOS) or 72 h (iNOS and eNOS) under continuous rotatory shaking. Then, the slices were washed with PBS (3 × 10 min) and incubated for 2 h at room temperature with either a Cy2-conjugated donkey anti-sheep (1:500 v/v; Jackson, Bar Harbor, ME, USA), Cy2-conjugated donkey anti-mouse (1:500 v/v; Jackson) or Cy3-conjugated donkey anti-mouse (1:500 v/v; Jackson) secondary antibodies in PBS, depending on the primary antibody used. Five minutes before removing the secondary antibodies, 4′,6-diamidino-2-phenylindole (DAPI; Sigma-Aldrich, Saint Louis, MO, USA) was added to each well until reaching a final dilution of 1:10000 to counterstain the cell nuclei. Finally, the sections were serially mounted on gelatinized slides, with a medium that prevented fluorescence from fading, and then covered with a coverslip for preserving the tissue.

To confirm that the nNOS labeling was present in neurons, some sections were subjected to a double immunohistochemistry technique by incubating them with sheep anti-nNOS and Cy2-conjugated donkey anti-sheep secondary antibodies, as explained above, together with rabbit anti-MAP2 primary antibody (1:150 v/v; Chemicon, Temecula, CA, USA) and Cy3-conjugated donkey anti-rabbit secondary antibody (1:500 v/v; Jackson; Supplementary Figure 1).

Subsequently, all sections were observed and analyzed using an epifluorescence microscope (Olympus Provis AX70, Japan) with 20X and 40X objectives. For the quantitative analyses, sections at three different and comparable rostro-caudal levels were selected (Allen, 2004). The section selected for examining the caudalmost level was the first one contiguous to the AOB without containing it (ca. Bregma 4.145 mm; Allen Brain Atlas) (Allen, 2004); the rostral level was set at the rostralmost section in which all MOB layers were clearly defined (Bregma 5.345; Allen Brain Atlas); and the medial level was defined as the midway section between the two previous ones (ca. Bregma 4.745; Allen Brain Atlas). First, the density of nNOS-positive cells was estimated in the whole area of the sections analyzed, focusing on the glomerular layer (GL) and the granule cell layer (GCL), where all satine cell profiles with at least a non-stained nuclear section and with at least one neurite nNOS stained were counted. The number obtained was then referred to the surface of the area analyzed. Regarding iNOS, no expression was found in the sections of the MOB analyzed in any of the experimental models; therefore, no further analyses were carried out (see Section “Results”). Concerning eNOS, its expression was restricted to endothelial cell-like elements that lined the blood vessels throughout the area of the different sections of the MOB analyzed. This labeling was semi-automatically measured considering both the tissue region occupied by the blood vessels (eNOS blood vessels density) and the intensity of their labeling (eNOS “labeling density”; see Section “Results,” Figure 4 and Supplementary Data 1); (Matos et al., 2016; Ferreiro et al., 2018; De Brito Robalo et al., 2020).

### Nicotinamide-adenine dinucleotide phosphate diaphorase activity

Nicotinamide adenine dinucleotide phosphate (NADPH) diaphorase activity was evaluated by measuring the enzymatic activity of NOS. The mice destined for NADPH diaphorase activity were the same as those destined for histological analyses (*n* = 4 for each group). MOB sections were incubated in a medium made up of PBS, nitro blue tetrazolium (Sigma-Aldrich), 0,2% triton X-100 (v/v), and β-NADPH (Sigma-Aldrich) for 60–120 min at 37°C. The sections were serially mounted on gelatinized slides and dehydrated in an ethanol battery of ascending graduation. Finally, the sections were covered with Entellan (Merck Millipore, Burlington, MA, USA) and a coverslip for their preservation. Subsequently, the sections were observed and analyzed using bright field microscopy (Olympus Provis AX70) and 10X, 20X, and 40X objectives. The activity of NADPH diaphorase was measured considering the density of the labeling. For this, all blood vessels positive for NADPH diaphorase were counted, and the resulting number was referred to the surface area analyzed.

### Biochemical analysis

The mice employed for this study (*n* = 9 for each group) were sacrificed by cervical dislocation and subsequent decapitation. The MOBs were extracted and homogenized in 200 μl of PBS, centrifuged at 1,000 *g* for 15 min at 4°C to remove insoluble material, and the supernatant was collected. The direct quantification of NO is complicated, as NO diffuses and reacts quickly with the surrounding environment (Csonka et al., 2015). Therefore, the levels of NO were estimated by quantifying both NO_3_^–^ and NO_2_^–^, the main NO metabolites. For this purpose, the nitrite/nitrate assay colorimetric kit was used (Sigma-Aldrich, Saint Luis, MO, USA). This kit allows the levels of NO metabolites to be determined using the Griess assay. From each sample, 40 μl of the supernatant containing both NO_3_^–^ and NO_2_^–^ were added, in duplicate, to a 96-well plate (Abcam, Cambridge, UK). During this process, NO_3_^–^ is converted to NO_2_^–^ by the enzyme nitrate reductase. Then, the Griess assay works by the azo coupling between diazonium species, which are produced from sulfanilamide and naphthylethylenediamine with NO_2_^–^. This reaction results in a colorimetric product proportional to the concentration of NO metabolites present in each sample. Once the reaction is complete, absorbance can be measured using a microplate reader at an absorbance of 540 nm (Thermo Fisher Scientific). The values obtained were referred to a standard line of known NO_2_^–^ concentrations, following the manufacturer’s instructions.

### Statistical analysis

All of the images obtained were analyzed using the open-source image analysis software Fiji (Schindelin et al., 2012). Data collection and classification were done using the programs Excel (v19.v, Microsoft Office, Albuquerque, NM, USA) and GraphPad Prism (v.8.0.0, Windows, GraphPad Software, San Diego, CA, USA). As two experimental groups were compared in all experiments, the statistical analyses conducted were performed using the Mann–Whitney’s U test, with the statistical package for the social sciences (SPSS) Statistics for Windows program (v.23.0; IBM, New York, NY, USA). Data were presented as Mean ± Standard Deviation (SD) and *p*-values lower than 0.05 were deemed to indicate statistical significance.

## Results

### nNOS-KO mice take longer to find buried food than wild-type mice

**FIGURE 1:**
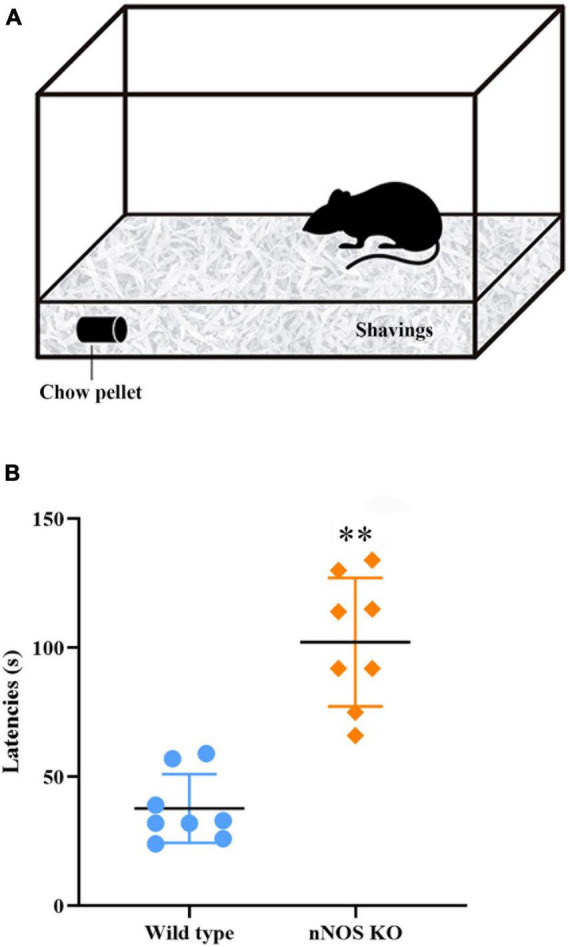
Buried food test. **(A)** Schematic representation of the device employed. **(B)** Chart showing the delay, in seconds, in the time it takes for the mice to find the buried piece of food. It was observed that nNOS-KO mice took longer to find the buried food compared to wild-type mice, with highly significant differences between both experimental groups (nNOS KO 94.87 ± 35.05 s; wild type 45.12 ± 23.06 s; *p* = 0.007; ^**^p < 0.01).

The results indicated that the absence of nNOS in KO mice interferes with their ability to smell as compared to wild-type animals. The buried food test showed that nNOS-KO mice take more time to locate the pellet of food than their wild-type counterparts, resulting in significant differences between both experimental groups (nNOS KO 94.87 ± 35.05 s; wild type 45.12 ± 23.06 s; *p* = 0.007; Figure 1).

It should be noted that during this test the nNOS-KO animals seemed to present more nervous and exploratory behavior than the wild type. The possible influence of such behavior on performance in the olfactory tests has been assessed by analyzing different movements of the mice during the test. Although the nNOS-KO mice seemed to move more while searching for the pellet, the statistical analysis revealed no significant differences between wild-type and nNOS-KO mice for the number of times they approached the pellet (nNOS KO 3.87 ± 2.25; wild type 2.25 ± 0.7; *p* = 0.105), the number of uprisings (nNOS KO 2.87 ± 1.88; wild type 1.5 ± 1.77; *p* = 0.161), and the number of groomings (nNOS KO 2.5 ± 1.51; wild type 1.12 ± 0.64; *p* = 0.105; Supplementary Figure 2).

Once the influence of the lack of nNOS on olfaction had been assessed, we decided to further investigate this effect. Since nNOS is the main olfaction-related isoform, its distribution along the rostro-caudal axis of the MOB in mice was examined by analyzing its distribution pattern.

### nNOS is expressed throughout the entire main olfactory bulb

First, we analyzed the distribution of nNOS in the MOB of wild-type mice. This preliminary study was aimed at determining in which bulbar regions and layers nNOS was normally expressed. This information would be used to identify possible changes that might occur in nNOS-KO mice concerning the expression and/or function of other NOS isoforms. In wild-type mice, the expression of nNOS was observed in different layers of the MOB. By contrast, and as expected, in nNOS-KO mice, nNOS protein was not detected (Supplementary Figure 3). In the case of wild-type mice, two main cell types expressing nNOS were found: in neurons in the GL with a spherical soma that generally possesses a single neurite; and in neurons in the GCL presenting a polygonal or spindle-shaped morphology with 2 or 3 main and sparsely branched neurites. Only a few cells positive for nNOS were found in the rest of the MOB layers. Consequently, the quantitative analysis of these cells was omitted.

**FIGURE 2:**
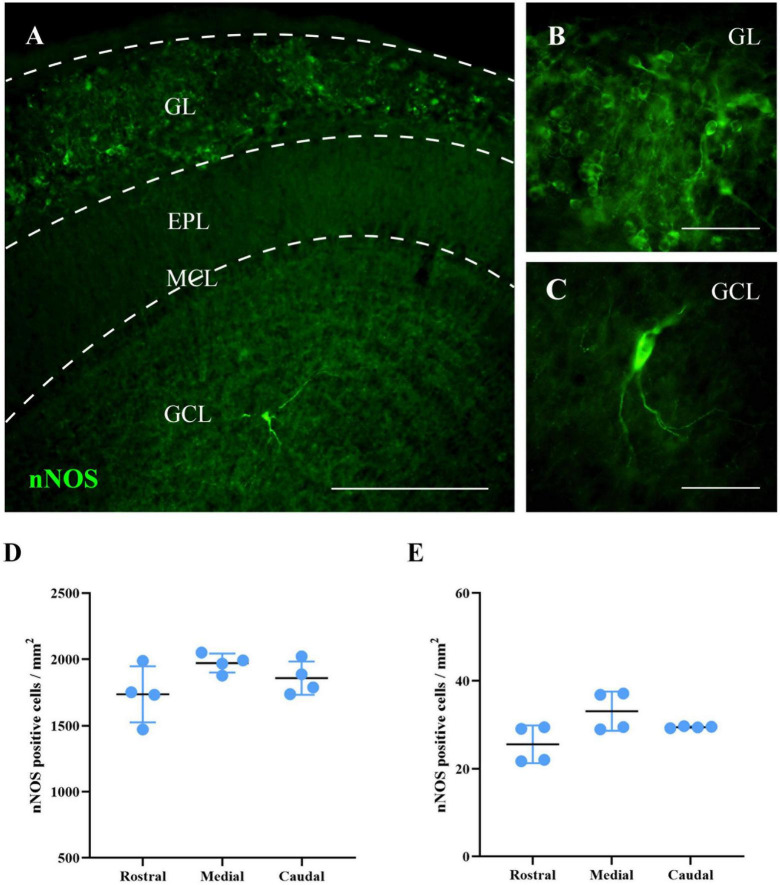
Labeling of nNOS in the MOB of wild-type mice. **(A)** Image showing a panoramic view of the nNOS labeling (green). **(B,C)** Magnifications of neurons labeled for nNOS in GL **(B)** and GCL **(C)**. **(D,E)** Charts summarizing the analyses of the density of cells positive for nNOS in the GL **(D)** and GCL **(E)**, in different levels of the MOB (rostral, medial, and caudal). The same density of nNOS positive cells per mm^2^ was observed throughout the entire area of the GL, with no significant differences among the levels analyzed (rostral 1,736 ± 211 cells/mm^2^, medial 1,971 ± 72 cells/mm^2^, caudal 1,858 ± 126 cells/mm^2^; *p* = 0.146) or the GCL (rostral 25 ± 4 cells/mm^2^, medial 33 ± 4 cells/mm^2^, caudal 29 ± 1 cells/mm^2^; *p* = 0.116). Scale bars 200 μm **(A)** and 100 μm **(B,C)**.

Then, the density of cells positive for nNOS in the GL and GCL was calculated at three different rostro-caudal levels. The same density of nNOS positive cells per mm^2^ was observed throughout the entire area of the GL, with no significant differences among the levels analyzed (rostral 1,736 ± 211 cells/mm^2^, medial 1,971 ± 72 cells/mm^2^, caudal 1,858 ± 126 cells/mm^2^; *p* = 0.146) or the GCL (rostral 25 ± 4 cells/mm^2^, medial 33 ± 4 cells/mm^2^, caudal 29 ± 1 cells/mm^2^; *p* = 0.116; Figure 2). These results indicate that the expression of nNOS in wild-type mice is constant throughout the rostral-caudal axis of the MOB for each bulbar layer.

Once the neuronal nature and distribution of nNOS-positive cells in wild-type mice were assessed, and their lack of detection was confirmed in the KO animals, the expression of iNOS and eNOS in the MOB of both experimental groups was analyzed.

### Gene and protein expression of iNOS and eNOS isoforms in wild-type and nNOS-KO mice

**FIGURE 3:**
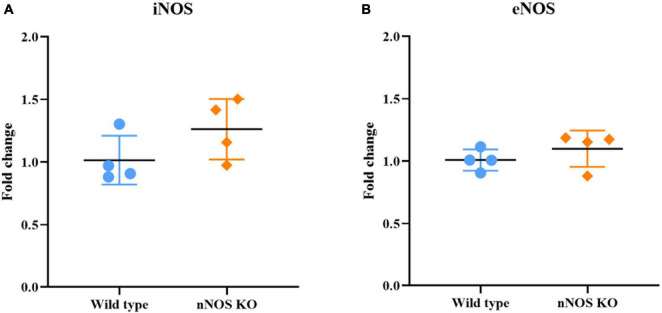
Genetic analyses of iNOS and eNOS. Charts summarizing statistical analyses of the iNOS **(A)** and eNOS gene expression **(B)** in the MOB of wild-type and nNOS-KO mice. The mRNA expression of genes corresponding to the iNOS and eNOS isoforms was similar for both experimental animal models, with no significant differences between the experimental groups (iNOS in wild-type mice 1.01 ± 0.19 fold change and iNOS in nNOS-KO mice 1.26 ± 0.24 fold change [*p* = 0.114; **(A)**]; eNOS in wild-type mice 1.01 ± 0.08 fold change and eNOS in nNOS-KO mice 1.09 ± 0.14 fold change [*p* = 0.886; **(B)**].

The qPCR analysis determined that the mRNA expression of genes corresponding to the iNOS and eNOS isoforms was similar for both experimental animal models, with no significant differences: iNOS in wild-type mice 1.01 ± 0.19 fold change and iNOS in nNOS-KO mice 1.26 ± 0.24 fold change (*p* = 0.114; Figure 3A); eNOS in wild-type mice 1.01 ± 0.08 fold change and eNOS in nNOS-KO mice 1.09 ± 0.14 fold change (*p* = 0.886; Figure 3B).

**FIGURE 4:**
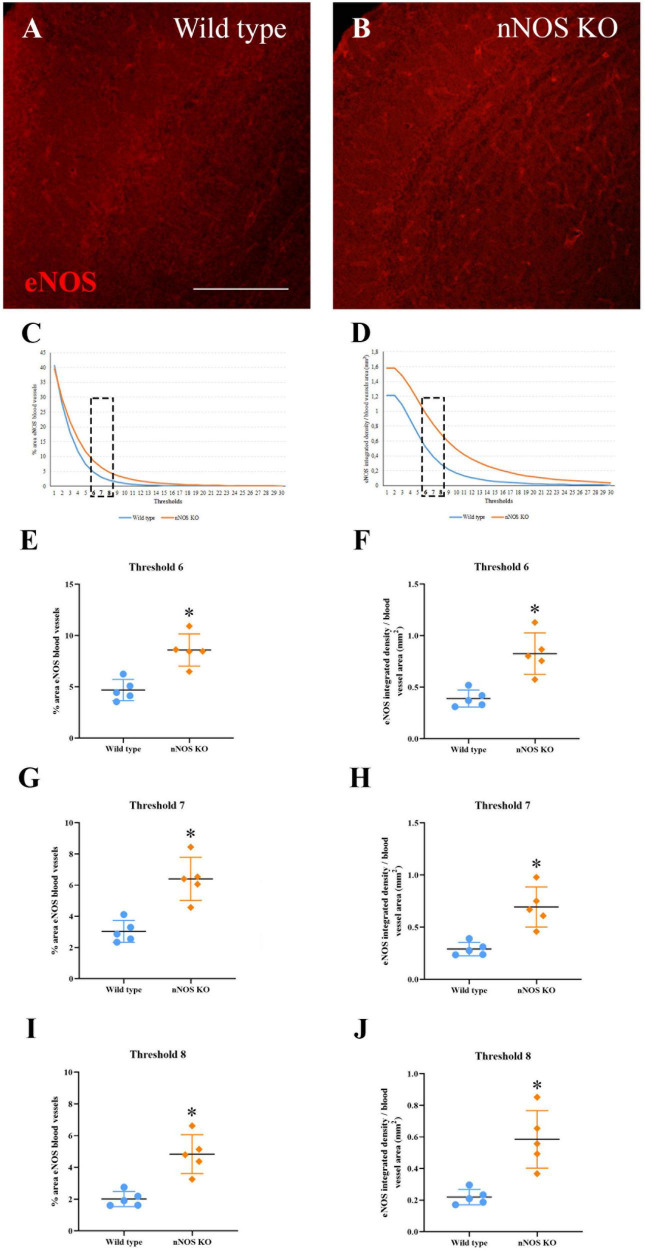
Expression of eNOS in the MOB. **(A,B)** Immunolabeling of eNOS (red) in blood vessels of wild-type **(A)** and nNOS-KO mice **(B)**. **(C,D)** Curves corresponding to the threshold values at different exposures for the area occupied by the labeled blood vessels **(C)** and the integrated density per area corresponding to eNOS **(D)**. **(E–J)** Graph representations summarizing statistical analyses of the percentage occupied by eNOS in the blood vessels **(E,G,I)** and eNOS integrated density per area **(F,H,J)** for the three thresholds analyzed (#6, #7, and #8). Note that in the three selected thresholds, there are significant differences between experimental groups for both parameters. *p < 0.05. Scale bar 200 μm.

Regarding the immunohistochemical analyses, iNOS expression was not observed in the MOB of either experimental group (Supplementary Figure 4). Conversely, eNOS expression appeared in the blood vessels of the MOB in both wild-type (Figure 4A) and nNOS-KO mice (Figure 4B). As explained previously, Fiji macro was used to select eNOS-labeled blood vessels and to quantify the mean surface area (Figure 4C), as well as the integrated intensity for the staining (Figure 4D). For both parameters, two curves were plotted corresponding to all of the exposure thresholds analyzed. The region of the curves where the differences among experimental groups were more evident corresponded to the thresholds of intermediate image exposure (Figures 4C, D). Three of these average thresholds (#6, #7, and #8) and their associated dependent values (for either mean surface or integrated density) were considered for further quantitative analyses (see Experimental Procedures; Figures 4C, D).

Upon considering the density (percentage of the area with stained blood vessels/surface analyzed). Our results showed there were significant differences in the three thresholds analyzed between the two experimental groups. For threshold #6, 4.69 ± 1.02% of the blood vessel density was estimated for wild-type vs. 8.59 ± 1.57% for nNOS-KO mice (*p* = 0.032; Figure 4E). For threshold #7, 3.03 ± 0.7% was calculated for wild-type vs. 6.4 ± 1.38% for nNOS-KO mice (*p* = 0.016; Figure 4G). Finally, for threshold #8, 2.01 ± 0.47% corresponded to wild-type vs. 4.84 ± 1.22% to nNOS-KO mice (*p* = 0.016; Figure 4I). Regarding the analysis of the staining intensity of eNOS, the results also showed there were significant differences in the three corresponding thresholds (integrated intensity/blood vessel density) between the experimental groups. For threshold #6, 0.39 ± 0.08 integrated intensity/mm^2^ in wild-type mice was obtained, in comparison to 0.82 ± 0.2 integrated intensity/mm^2^ in nNOS-KO animals (*p* = 0.016; Figure 4F). For threshold #7, 0.29 ± 0.06 integrated intensity/mm^2^ was obtained in wild-type mice vs. 0.69 ± 0.19 integrated density/mm^2^ in nNOS-KO mice (*p* = 0.008; Figure 4H). For threshold #8, 0.21 ± 0.04 integrated density/mm^2^ was obtained in wild-type mice vs. 0.58 ± 0.18 integrated density/mm^2^ in nNOS-KO mice (*p* = 0.008; Figure 4J).

These results showed that in nNOS-KO mice there is a higher expression of the eNOS isoform, which may be due to a compensatory modification in the absence of nNOS. To confirm this assumption, the activity of NADPH diaphorase as an estimator of eNOS functioning was studied.

### Nicotinamide-adenine dinucleotide phosphate diaphorase activity is more intense in the blood vessels of nNOS-KO mice

We performed the NADPH diaphorase test to analyze the activity of NOS. In the central nervous system, this technique reveals diverse populations of neurons and blood vessels that express some of the NOS isoenzymes (Weruaga et al., 1998; Csonka et al., 2015). In our case, in wild-type mice, the NADPH diaphorase activity was found in neurons located mainly in both GL and GCL, as well as in the blood vessels of the entire MOB. Qualitatively, the labeling intensity was higher in neurons (corresponding to nNOS) than in blood vessels (corresponding to eNOS) thus making these two kinds of staining perfectly distinguishable.

**FIGURE 5:**
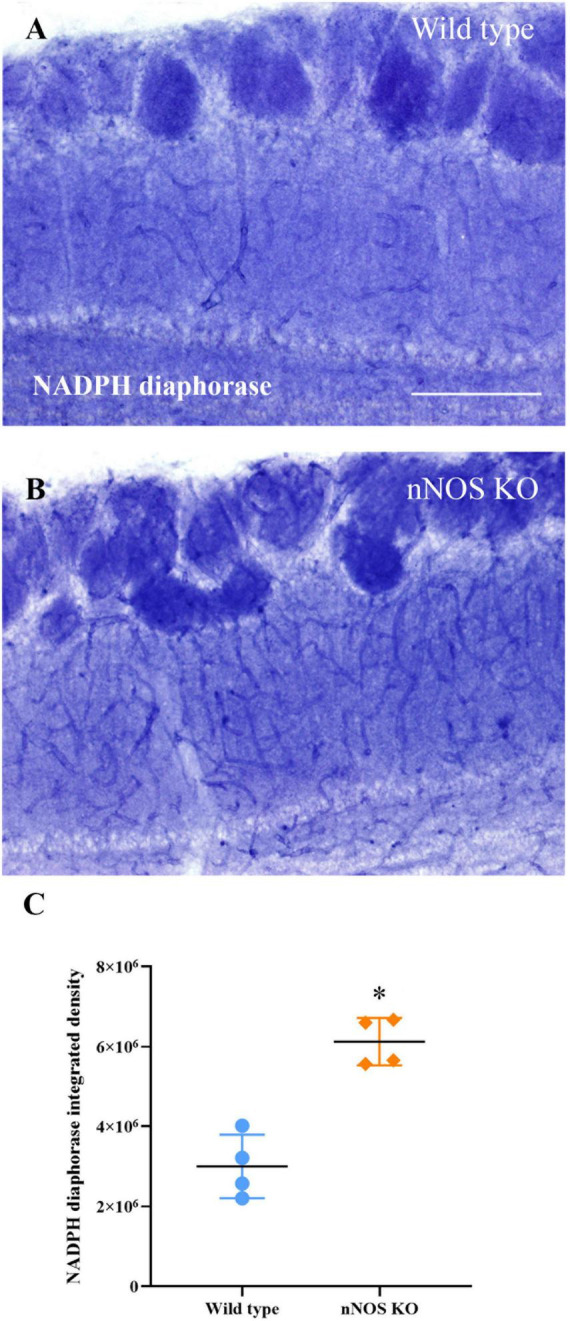
Analysis of NADPH diaphorase activity. Labeling of NADPH diaphorase in blood vessels of wild-type **(A)** and nNOS-KO mice **(B)**. **(C)** Chart showing the analysis of NADPH diaphorase staining present in the blood vessels of both animal models; note that nNOS-KO mice show more intense labeling in comparison with the wild-type animals (wild type 2999976.75 ± 796295.55 integrated density/mm^2^; nNOS KO 6126408.75 ± 592378.10 integrated density/mm^2^; *p* = 0.029). *p < 0.05. Scale bar 200 μm.

Regarding the labeling found in the nNOS-KO mice, NADPH diaphorase activity was found only in vascular structures. Therefore, we compared only the labeling intensity corresponding to blood vessels between groups. We determined that the intensity of the labeling was significantly higher in the nNOS-KO mice (wild type 2999976.75 ± 796295.55 integrated density/mm^2^; nNOS KO 6126408.75 ± 592378.10 integrated density/mm^2^; *p* = 0.029; Figure 5).

Based on this finding, the total levels of NO_3_^–^ and NO_2_^–^ (main NO metabolites) produced in the MOB of both animal models were measured. With this experiment, we aimed to verify if the increase in eNOS activity in nNOS-KO mice was able to equalize the levels of NO produced in wild-type animals.

### The global production of nitrates and nitrites is similar in wild-type and nNOS-KO mice

**FIGURE 6:**
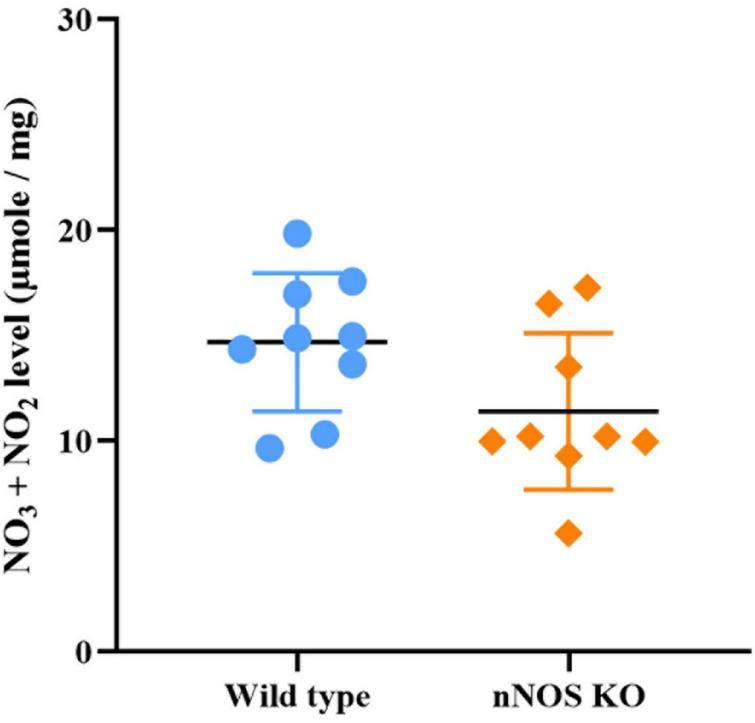
Concentration of stable NO metabolites in the MOB in wild-type and nNOS-KO mice. No significant differences between experimental models were found (wild type 14.68 ± 3.55 μmole/mg; nNOS KO 11.52 ± 3.88 μmole/mg; *p* = 0.281).

Although the production of NO_3_^–^ and NO_2_^–^ appeared slightly higher in wild-type mice, no significant differences between both experimental groups were found for this parameter (wild type 14.68 ± 3.55 μmole/mg; nNOS-KO 11.52 ± 3.88 μmole/mg; *p* = 0.281; Figure 6).

Therefore, considering the results obtained, we can affirm that even though KO mice do not present nNOS (the main NO-producing enzyme in the nervous system), their NO concentration is similar to that of wild-type animals. This result suggests there is a compensatory modification in NO production by eNOS in nNOS-KO mice that is probably aimed at maintaining optimal NO levels throughout the MOB.

## Discussion

Currently, it is known that both the MOB and NOS show great plasticity, which helps rodents to adapt to various pathological or abnormal conditions (Khan et al., 2012; Sung et al., 2014; Zou et al., 2014; Muñoz et al., 2015; Díaz et al., 2017); however, not much is known about the plasticity of NOS in the MOB itself. For this reason, we studied the plasticity of NOS and the production of NO, and possible olfactory alterations in an animal model lacking nNOS, the main encephalic isoform, to observe the behavior of the remaining types of NOS.

Initially, we analyzed whether the lack of nNOS affected the correct physiology of the MOB, as nNOS plays an important role in processes related to memory, olfactory learning, and the uptake of odorant molecules (Dinerman et al., 1994; Crespo et al., 2003; Kosaka and Kosaka, 2007; Zou et al., 2014; Marvasi, 2017). To do this, the buried food test was conducted for studying the olfactory capacity of nNOS-KO mice. Our results indicate that the absence of nNOS compromises the olfaction of these animals because they take longer to find hidden food than wild-type mice do. These findings are in line with previous works demonstrating that the decrease in NO produced by nNOS influences olfactory capacity and other cognitive performances in mice (Weitzdoerfer et al., 2004; Lundberg et al., 2008; Jüch et al., 2009). In addition, as detailed below, nNOS-KO mice show an increased expression of the enzyme eNOS. This change could also affect the olfactory capacity of these animals since previous studies show that the altered expression of eNOS in the MOB can contribute to changes in behavior and olfaction (Brunert et al., 2009; Endo et al., 2011; Sung et al., 2014). Precisely, it should be noted that the behavior of wild-type and nNOS-KO mice during the buried food test differed. While wild-type animals sniffed calmly, pausing occasionally to identify the source of the odor, the nNOS-KO mice constantly and nervously moved around the cage until they found the piece of food. This disrupted behavior also could interfere with the execution of the buried food test. To discard this additional influence on pure olfactory impairments, we analyzed other behavioral parameters related to movement (i.e., approaching the pellet, uprising, and grooming). As no significant differences were detected between experimental groups, it seems that the absence of nNOS directly affects olfaction in mice. Other published work (James et al., 2015) describes that nNOS-KO mice show better olfactory learning and increased locomotor ability compared to wild-type animals. Apparently, these results seem to contradict ours, both for the buried food test and in the movement analyses. However, in the above-mentioned research, olfactory learning was assessed, whereas we analyzed the pure olfactory ability to capture volatile odors (Yang and Crawley, 2009) without any prior learning (in fact, our test was only performed once). Concerning locomotor activity, in the previous work the authors report an age-related effect where only young nNOS-KO animals were significantly more active than wild-type mice but not the old animals (James et al., 2015). Nevertheless, the ages analyzed and the parameters measured in our work differ notably from those of the previous research. In this sense, although a certain influence of movement cannot be completely discarded, at least the parameters assessed in our work seem not to affect the olfactory test employed. Therefore, the absence of nNOS impairs, at least partially, the olfactory performance of mice.

The next experiment consisted of analyzing the standard distribution of nNOS throughout the entire rostro-caudal axis of the wild-type MOB. Agreeing with previous studies (Csonka et al., 2015), extremely high expression of nNOS was found in GL cells and low expression in GCL. Given the MAP2 labeling of these elements, and considering their morphology and the existing bibliography, we can affirm that the marking of nNOS-positive cells in the mice used in this study corresponds to different types of neurons (Kishimoto et al., 1993; Dinerman et al., 1994; Yand et al., 2013). Considering the specific type of neurons, it is not easy to classify them considering their neurochemical nature. Thus, many immunohistochemical procedures should be performed to completely characterize these cells, as well as those located in different OB layers. This in-depth characterization has been already addressed by other authors (Crespo et al., 2003; Kosaka and Kosaka, 2007) and is clearly beyond the scope of the present work. Additionally, we found the same density of nNOS-positive cells in the GL and the GCL layers throughout the entire MOB (rostral, medial, and caudal levels). This could indicate that the synthesis of NO by nNOS is essential for the neuronal organization of the MOB in the adult mouse, influencing both cell turnover and the functioning of this organ (Kishimoto et al., 1993; Dinerman et al., 1994; Yand et al., 2013; Sung et al., 2014; Marvasi, 2017; Price and Banks, 2017).

Likewise, we also performed genetic and immunohistochemical analyses for the other two NOSs to determine whether the lack of nNOS in KO mice influenced the expression of the other isoforms. The results concerning the genetic and immunohistochemical study of iNOS are coherent since this is an inducible enzyme that is mainly expressed in pathological and tissue damage conditions (Kendrick et al., 1997). For example, in the BALB/c mouse model infected with influenza A/NWS/33, only iNOS expression was observed, while no differences in nNOS or eNOS levels were observed between infected and uninfected mice (Watanabe et al., 2008). Moreover, although nNOS-KO mice present an absence of the nNOS enzyme, this fact does not trigger *a priori* any harmful condition in the MOB (Zou et al., 2014). It is true that in other tissues, such as dental pulp and odontoblasts, nNOS-KO mice undergo an increase in the expression of iNOS thus changes in its expression seem to be linked to tissue type. That is to say, the expression of one NOS isoform or another would be determined by the physiological needs of the tissue where it is found, possibly to compensate for any decrease in NO and to maintain optimal NO levels (Bolaños and Almeida, 1999; Zhu et al., 2003; Herbert et al., 2012; Khan et al., 2012; Muñoz et al., 2015; Ishizuka et al., 2019).

In this line of reasoning, the result of the expression analysis of the last isoform analyzed, eNOS, is noteworthy, as its expression was observed in both wild-type and the nNOS-KO mice. However, the level was much higher in the latter, not only in surface area but also in the intensity of the labeling.

It could be possible that the increase in the abundance of eNOS in mutant mice is in response to the absence of nNOS, as a compensatory mechanism. The function of nNOS is not always completely neuronal (Price and Banks, 2017; Ishizuka et al., 2019), because nNOS also participates in vasodilation processes that provide adequate blood flow (Muñoz et al., 2015; Lourenço et al., 2017). Similarly, apart from its main function, vasodilation, eNOS performs other functions neuronal in nature such as participating in synaptic plasticity and neuronal regulation and survival (Costa et al., 2016; Caviedes et al., 2017). But the data concerning eNOS gene expression (similar for both two experimental models) are somewhat disconcerting. One possible explanation is that the increase in the protein level of eNOS could be due to an increase in its translation from mRNA (Esplugues, 2002; Reber et al., 2002). Therefore, although we did not find differences between the two animal models at the level of gene expression, the increase in the translation of mRNA to protein in nNOS-KO mice would produce higher levels of eNOS, as we observed in our results. In fact, previous studies have shown that once mRNA is synthesized, eNOS exhibits strong regulation at the post-transcriptional and post-translational levels (Kishimoto et al., 1993; Ishizuka et al., 2019). Regarding post-transcriptional regulation, modifications of the mRNA occur first, to grant its stability and the subcellular location that the enzyme will adopt. Lastly, there is an additional translational regulation that depends on the processes of activation by acetylation, protein-protein interaction, phosphorylation, and binding of calcium with calmodulin (Oliveira-Paula et al., 2016; Zhu et al., 2016). Furthermore, the regulation of this enzyme does not end here; eNOS is primarily synthesized in the form of a monomeric enzyme and is inactive (Iwakiri, 2015; Oliveira-Paula et al., 2016). For it to be activated, the union of two monomers has to be produced, so that in the nNOS-KO mice, more eNOS monomers could bind, making the protein more active in these animals compared to the wild type.

To corroborate this hypothetical compensation, the level of NOS activity was also estimated by measuring NADPH diaphorase activity. In our analyses, NADPH diaphorase labeling was found in both GL and GCL neurons, and the blood vessels supplying the MOB. Its labeling was much higher in neurons (Kishimoto et al., 1993) since the nNOS isoform is the main NO producer in MOB. Precisely, in the nNOS-KO mice, we only found NADPH diaphorase labeling in the blood vessels. When we compared the vascular labeling in both experimental models, we observed that the intensity of NADPH diaphorase was higher in the nNOS-KO mice than in wild-type animals. Therefore, these data suggest that in nNOS-KO mice the eNOS present in MOB is more active, producing more NO than in wild-type mice. All these results could suggest that nNOS-KO mice have an increased vasculature compared to wild-type mice. Indeed, other studies describe increased angiogenesis and vasculature in situations of increased eNOS expression (Namba et al., 2003), which fits with our results. Therefore, this increase in both eNOS protein expression and activity in nNOS-KO mice may occur to compensate for the lack of nNOS, thus producing NO similar levels to the wild type (Kendrick et al., 1997; Huang et al., 2004; Khan et al., 2012; Muñoz et al., 2015; Caviedes et al., 2017; Lourenço et al., 2017).

To verify this hypothesis, we measured NO production in the MOB in the two animal models. As similar values were found in both study models, such compensation was confirmed. However, eNOS expression was observed across all layers of MOB instead of one specific stratum, as is the case for nNOS expression. Based on these findings, it could be assumed that the increase in the expression of eNOS and the related NADPH diaphorase activity in nNOS-KO mice may have a global compensatory character, in an attempt to maintain the correct physiology of the MOB in response to the absence of nNOS in both neuronal and hemodynamic processes. However, it should be noted that despite this compensatory modification produced by eNOS, the olfactory capacity of nNOS-KO animals is impaired, probably because this physiological activity requires a more refined NOS activity, which can only be provided by the neuronal isoform.

## Data availability statement

The original contributions presented in this study are included in the article/Supplementary material, further inquiries can be directed to the corresponding authors.

## Ethics statement

This animal study was reviewed and approved by the Bioethics Committee of the University of Salamanca (reference numbers: #00613).

## Author contributions

DP-B, DD, and EW conceived the study and designed the experiments. DP-B, CH-P, and VC performed the experiments. DP-B, JV, JA, DD, and EW interpreted the data. DP-B was a major contributor in writing the manuscript and organizing the figures. All authors critically revised and approved the final manuscript.

## Footnotes

1
http://jaxmice.jax.org
