# Supplementary material for: The eNOS isoform exhibits increased expression and activation in the main olfactory bulb of nNOS knock-out mice
Source: Front Cell Neurosci. 2023 Mar 16;17:1120836. doi: 10.3389/fncel.2023.1120836 (PMC10061100; doi:10.3389/fncel.2023.1120836)
Supplement: Supplementary file 1 [file Data_Sheet_1.docx]

Supplementary Material

# Supplementary Data 1

For the eNOS protein analysis, a macro in the Fiji program (distribution of the open-source software ImageJ, specific to the analysis of biological images; Schindelin et al., 2012; 10.1038/nmeth.2019) was created to select the blood vessels labelled with eNOS in the digital microphotographs and to quantify both parameters: the mean surface area containing the labelled blood vessels and the “integrated labelling density of eNOS. Briefly, as has been done in previous work [37- 39], all possible exposure thresholds were established for both measurements: the first threshold was set at the point where no labelling was observed and the last where the images looked fully saturated. Finally, for all thresholds analysed, a value was obtained for the extent of eNOS labelling (area) and the intensity of eNOS labelling (integrated density) for blood vessels. For the graphical analyses, only intermediate threshold values were considered, corresponding to images with an average saturation of staining. For the statistical analysis, thresholds #6, #7, and #8 were selected, where the difference between the labelling curves of both experimental groups was more pronounced (see Results and Figure 4).

# Supplementary Figures

## Supplementary Figure 1

Immunolabelling for nNOS (green) and MAP2 (red) in the GL of MOB in wild-type mice. Images show the co-expression of both markers (arrowheads) demonstrating that nNOS is expressed in neurons. For better visualization of the labelling of both antibodies, MAP2 (B) and nNOS (C) are shown separately. Scale bars 200 µm.

## Supplementary Figure 2

Analysis of movement during the buried food test. Three different parameters were evaluated: the number of times the mice approached to the area where the pellet was buried (A), the number of uprisings (B) and the number of grooming events (C). In none of the three cases significant differences were found: number of pellet approaches (nNOS KO 3.87 ± 2.25; wild type 2.25 ± 0.7; p = 0.105; Supplementary Figure 2A), number of uprisings (nNOS KO 2.87 ± 1.88; wild type 1.5 ± 1.77; p = 0.161; Supplementary Figure 2B) and number of groomings (nNOS KO 2.5 ± 1.51; wild type 1.12 ± 0.64; p = 0.105; Supplementary Figure 2C).

## Supplementary Figure 3

Immunolabelling for nNOS (green) in the MOB of nNOS-KO mice. As expected, no nNOS expression was observed in the entire MOB in any of the layers. Nuclei are counterstained with DAPI (blue, B). Scale bars 200 µm.

## Supplementary Figure 4

Immunolabelling for iNOS (green) in the MOB of wild-type and nNOS-KO mice. No expression was observed in either wild-type (A, C) or nNOS-KO mice (B, D). Nuclei are counterstained with DAPI (blue, B, D) for a better visualization of the sections. Scale bars 200 µm.
