# Supplementary figures and images for: The eNOS isoform exhibits increased expression and activation in the main olfactory bulb of nNOS knock-out mice
Source: Front Cell Neurosci. 2023 Mar 16;17:1120836. doi: 10.3389/fncel.2023.1120836 (PMC10061100; doi:10.3389/fncel.2023.1120836)

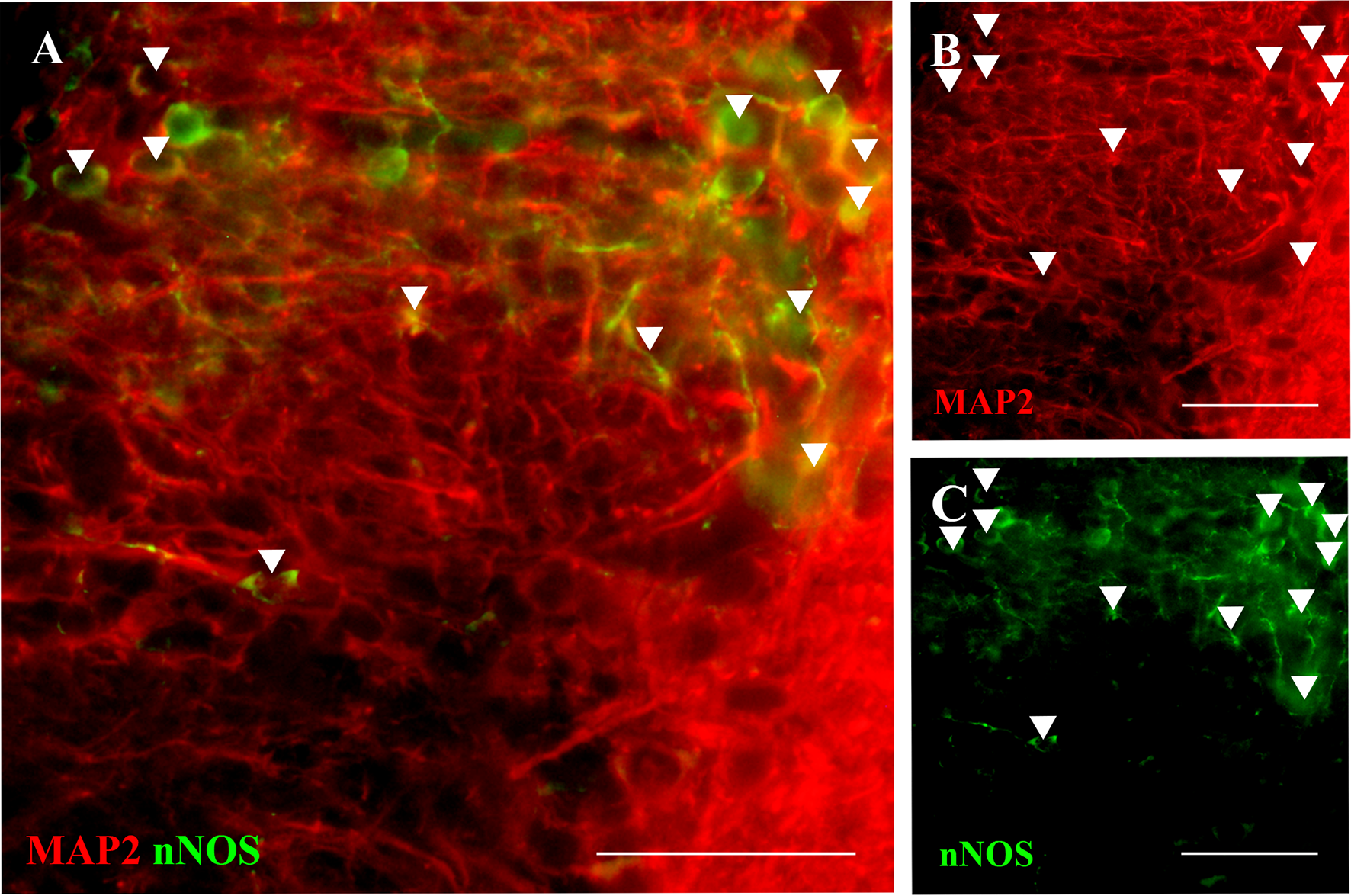

Supplement: Supplementary file 2 [file Image_1.TIF]
